# Light chain deposition disease involving kidney and liver in a patient with IgD myeloma

**DOI:** 10.1186/s12882-021-02246-9

**Published:** 2021-01-23

**Authors:** Takafumi Tsushima, Tomo Suzuki, Toshiki Terao, Daisuke Miura, Kentaro Narita, Masami Takeuchi, Akira Shimuzu, Kosei Matsue

**Affiliations:** 1 Division of Hematology/Oncology, Department of Internal Medicine, Kameda Medical Center, 929 Higashi-chou, Kamogawa-shi, Chiba, 296-8601 Japan; 2 Division of Nephrology, Department of Medicine, Kameda Medical Center, Kamogawa-shi, Chiba, 296-8601 Japan; 3 Department of Analytic Human Pathology, Nippon Medical School, Tokyo, Japan

**Keywords:** IgD myeloma, Light chain deposition disease, Liver involvement, Daratumumab, CyBorD

## Abstract

**Background:**

IgD multiple myeloma (MM) is a rare subtype of MM and light chain deposition disease (LCDD) outside the kidney is also a rare and has scarcely been reported. We report herein the details of the first reported case of LCDD involving the kidney and liver co-occurring with IgD myeloma.

**Case presentation:**

A 66-year-old female with IgD MM presented with rapidly progressive acute renal failure, ascites and pleural effusion. Immunofluorescent study of revealed the characteristic linear deposition of Ig*κ* light chain along the glomerular and tubular basement membrane in kidney. Electron microscopy showed the powdery electron-dense deposits along the tubular and glomerular basement membrane consistent with the diagnosis of LCDD. Laser microdissection followed by mass spectrometry identified only Igκ light chain with more than 95% probability confirm the diagnosis of κ-LCDD but not heavy/light chain deposition disease. Liver biopsy with immunofluorescence study revealed the linear deposition of Igκ chain along the perisinusoidal space indicating the hepatic involvement of κ-LCDD. The patient was successfully treated with combination therapy with bortezomib, cyclophosphamide, dexamethasone, and daratumumab.

**Conclusions:**

This report emphasizes that prompt biopsy of affected organs and initiation of clone directed therapy led to the correct diagnosis and favorable outcome in patient with LCDD who has extrarenal involvement.

**Supplementary Information:**

The online version contains supplementary material available at 10.1186/s12882-021-02246-9.

## Background

Light chain deposition disease (LCDD) is a relatively rare disease characterized by deposition of monoclonal either κ or λ immunoglobulin (Ig) light chain in various tissues [1–3]. Most of the patients with LCDD have plasma cell dyscrasia as an underlying disease. Involvement of LCDD in the kidney, liver, and heart often leads to respective organ failure and but is difficult to diagnose in routine clinical practice because of the need to demonstrate monoclonal immunoglobulin (Ig) deposition in biopsied samples by immunofluorescence (IF) and/or electron microscopy [4–6]. Therefore, despite the possible involvement of various organs, involvement of LCDD outside the kidney has scarcely been reported and most of reports have been case reports. In addition, the clinical scenario of LCDD outside of renal involvement remains obscure.

IgD myeloma is also a rare disease occurring in approximately 2–3% of patients with multiple myeloma (MM) [7, 8] It is characterized by a relatively young age of presentation, frequent renal complications, an aggressive clinical course, and predominantly involves the λ light chain type. Because of the rarity of both diseases, IgD MM complicating the LCDD has rarely been reported [9]. Further, liver involvement of LCDD in patients with IgD MM has not been previously reported.

Here, we report a patient with IgD MM who presented with acute renal failure and ascites. Kidney and liver biopsies revealed the LCDD of the aforementioned organs. The patient was successfully treated with daratumumab containing chemotherapy. To the best of our knowledge, this is the first report of a patient with IgD MM complicated by renal and hepatic LCDD.

## Case presentation

**Fig. 1:**
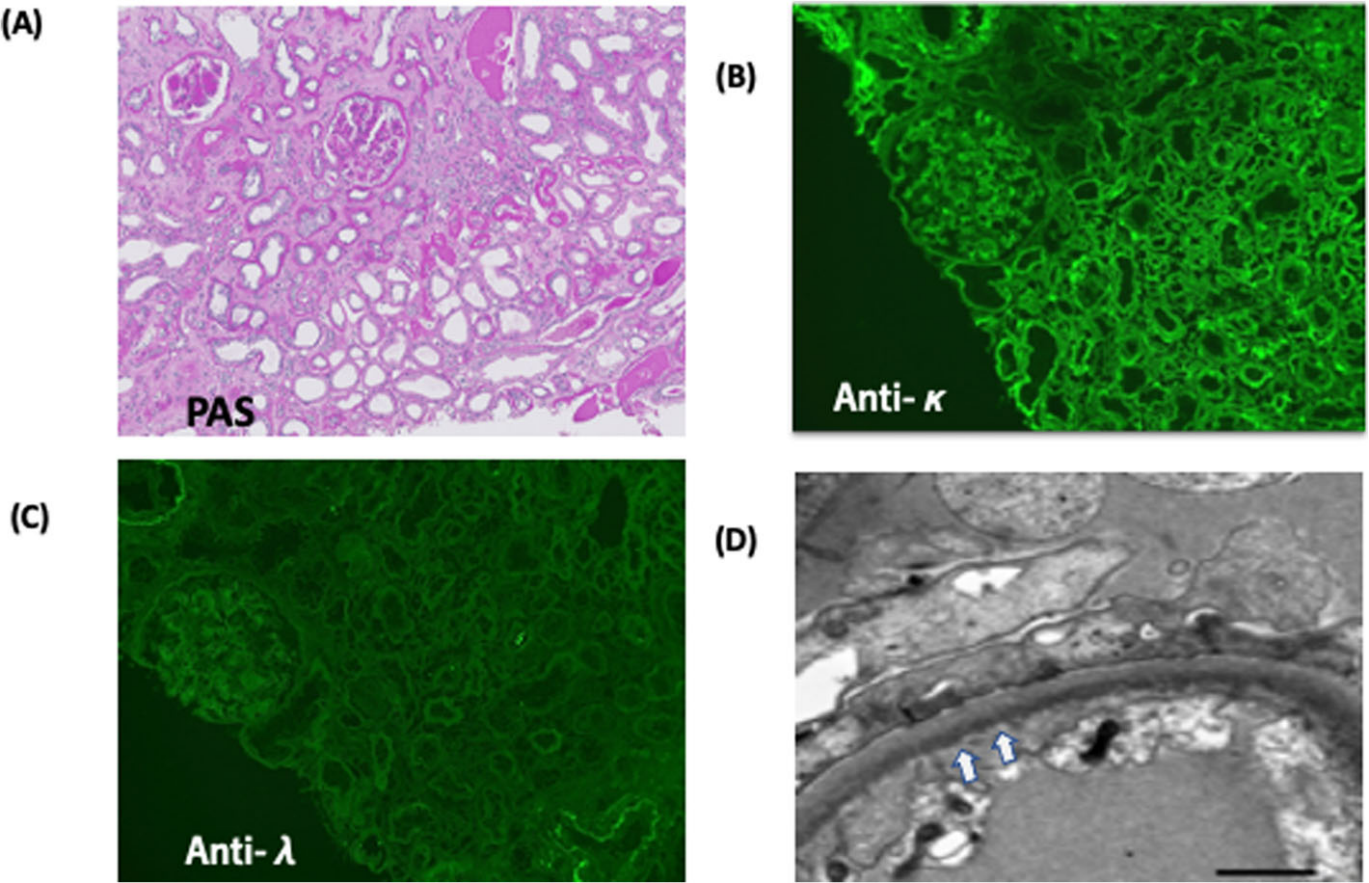
Histopathologic pictures of kidney biopsy. Light microscopy showed **a** nodular glomerular sclerosis by Periodic acid Schiff stain showed the positive staining of glomerular nodules and thickening of glomerular and tubular basement membranes (× 100). **b**, **c** Immunofluorescence showed positive staining for Igκ and negative staining for Igλ (× 100). **d** Electron microscopy showed powdery electron dense deposit along the glomerular and tubular basement membranes (arrow head) (× 6000)

**Fig. 2:**
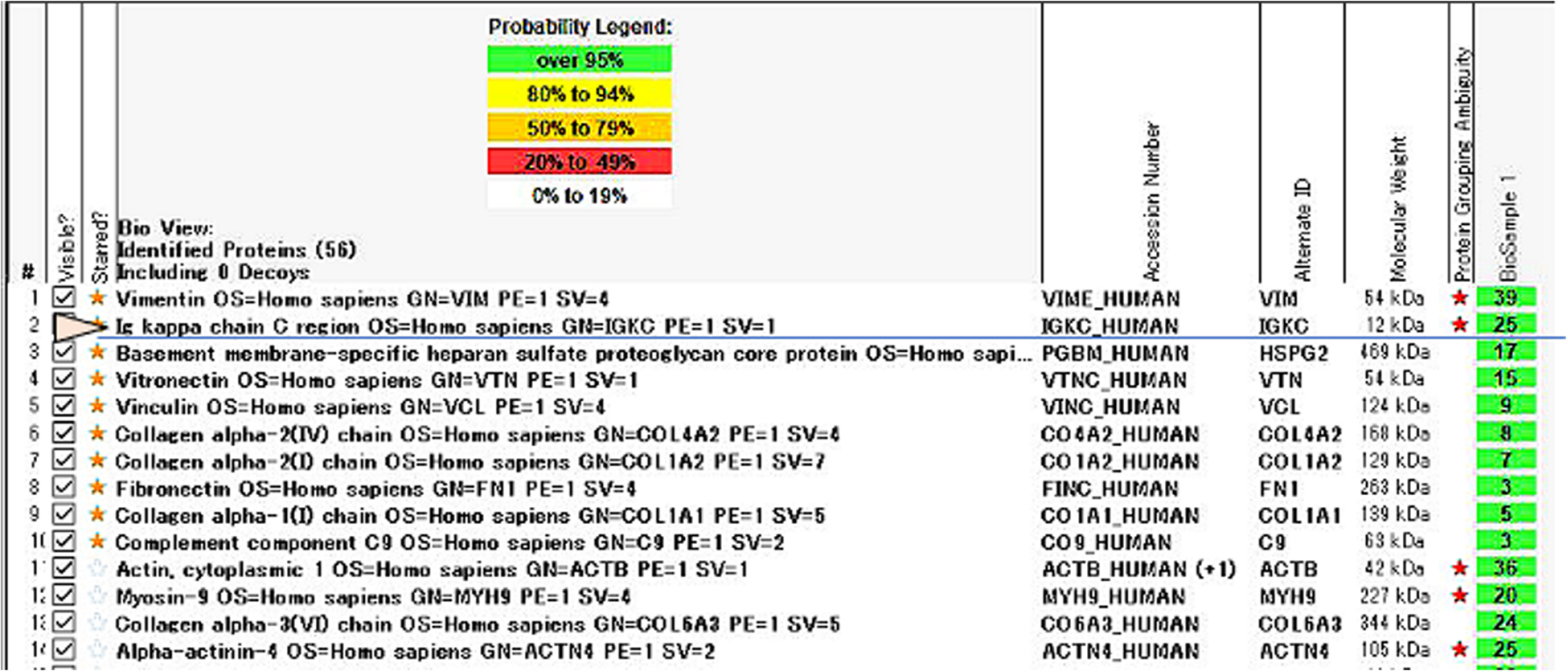
Results of laser microdissection followed by mass spectrometry (LMD/MS). LMD/MS Scaffold (Proteome Software Inc., Portland, OR, USA) identified 25 spectra numbers for Ig-kappa chain C region with more than 95% probability. Ig heavy chain, Ig-delta (IgD), and Igλ chain could not be detected, indicating the diagnosis of pure Igκ light chain deposition disease

**Fig. 3:**
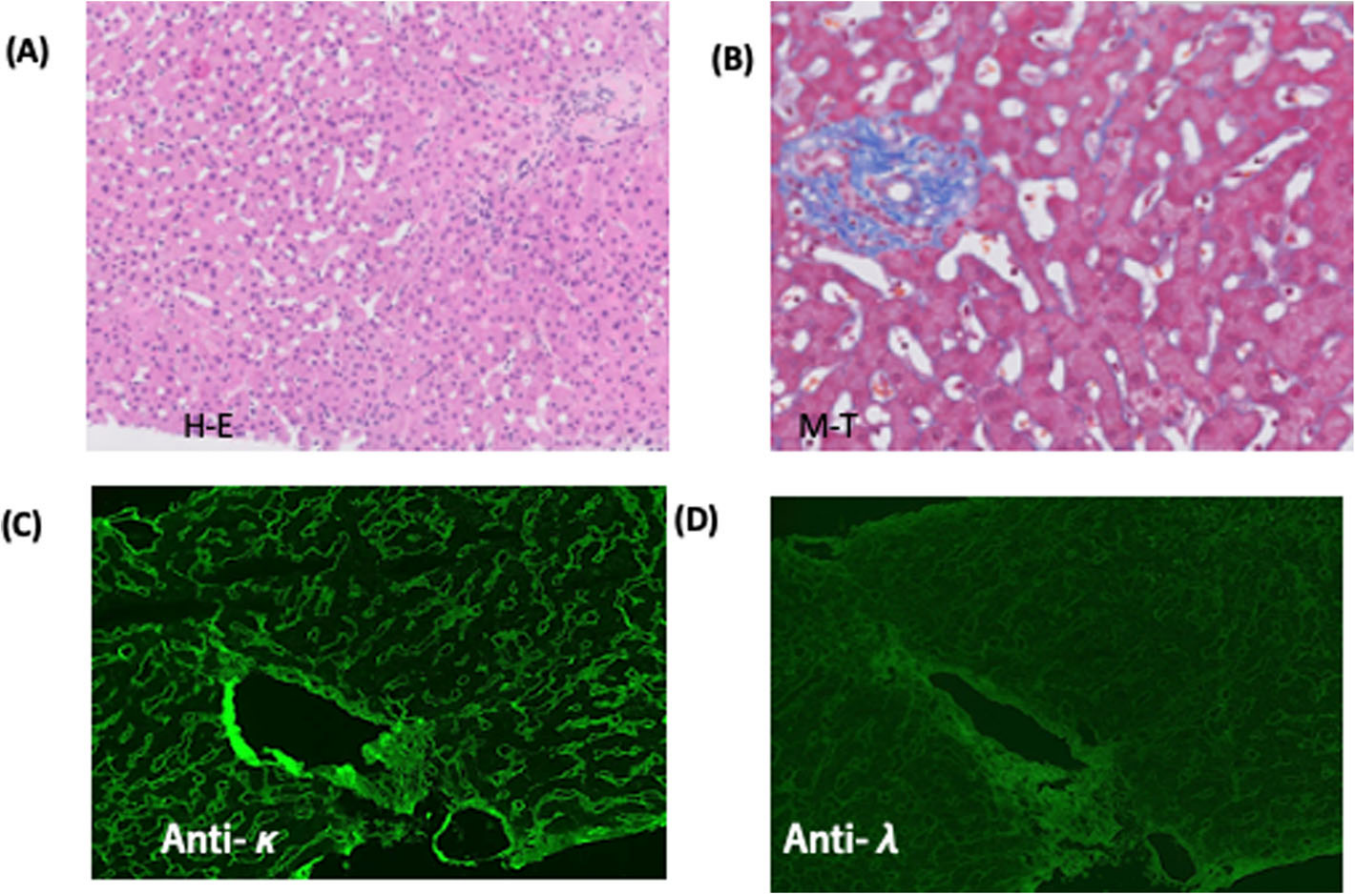
Hematoxylin-eosin staining (H-E) of liver biopsy showed the atrophy of hepatocytes and dilatation of sinusoidal space (× 100) (**a**). Masson-trichrome staining (M-T) showed the blue deposits along the perisinusoidal space (× 200) (**b**). Immunofluorescence showed positive for anti-Igκ (**c**) and negative for anti-Igλ (**d**) along the sinusoidal space and hepatic vein (× 100)

A 64-year-old Japanese woman was referred to us for the treatment of acute renal failure and elevated of free light chain κ (FLCκ, 6900 mg/L, normal, 2.42–18.92 mg/L). Her past medical history was unremarkable except for treatment of hypertension. One week before her referral, she was admitted to the local hospital for malaise and anorexia. Laboratory examination revealed anemia, (hemoglobin 9.2 g/dL) increased serum creatinine (5.6 mg/dL; normal, 0.6–1.2 mg/dL) and hypogammaglobulinemia (IgG 385 mg/dL; normal 680–1626 mg/dL, IgA 59 mg/dL; normal 84–438 mg/dL, IgM 5 mg/dL; normal 57–280 mg/dL)). She had 2+ urine protein by dipstick and urine protein was 1800 mg/day with 72.8% of albumin. Hematuria was negative. Hepatitis A, B, and C viruses and HIV were negative. The tentative diagnosis was acute renal failure due to glomerulonephritis of unknown cause. She was treated with 1 mg/kg of prednisone, but her symptoms had worsened. On admission to our hospital, mild ascites, pleural effusion, and enlarged both kidneys (13 × 7 cm) were noted by whole-body computed tomography (CT). Whole body diffusion weighted magnetic resonance imaging detected a focal plasmacytoma in the left jaw. Laboratory findings (Table 1) showed marked elevation of serum creatinine (6.31 mg/dL), free light chain κ (763.0 mg/L), and N-terminal pro-brain natriuretic peptide (NT-proBNP, 3643 pg/mL; normal, 0–125 U/mL) levels. Liver function test were normal, including aspartate aminotransferase (AST) alanine aminotransferase (ALT), and alkaline phosphatase were normal. Urinalysis showed proteinuria of 1800 mg/day with 73% of albumin. Serum protein electrophoresis (SPEP) showed a a small notch in the γ-lesion. Serum and urine immunofixation (IFx) revealed a faint monoclonal IgD*κ* band and a clear *κ*-light chain band, respectively. Serological studies for anti-nuclear antibody was negative and complement levels (C3 and C4) were within normal limits. Bone marrow biopsy showed 19% of plasma cell infiltration by CD138 immunostaining. Multicolor flow cytometry showed that 5% of monoclonal plasma cells those were restricted to *κ* light chain and were positive for CD56 and negative for CD19. Cytogenetic abnormalities including del17p, t (11;14), t (4;14), t (14;16), and del13p, and 1q gain were negative by fluorescence in situ hybridization (FISH) using the CD138 purified bone marrow cells. A kidney biopsy revealed the nodular glomerulosclerosis and casts by light microscopy (Fig. 1a). Congo red staining was negative. An IF study demonstrated the positive staining of Igκ (Fig. 1b) and negative staining of Igλ (Fig. 1c) light chain along the glomerular and tubular basement membranes. Immunohistochemistry showed linear staining of glomerular basement membrane positive for Igκ and negative staining for Igλ. Ribbon-like deposits by periodic acid Schiff (PAS) staining. Tubular casts positive for Igκ and negative for PAS and Igλ were also seen (Supplement figure, a-f). Electron microscopy showed the powdery electron-dense deposits along the tubular and glomerular basement membrane (Fig. 1d, arrow head) consistent with the diagnosis of LCDD involving the kidney. To determine the glomerular deposits, laser microdissection (LMD) of the of the glomerulus of paraffin-embedded kidney biopsy material followed by liquid chromatography and mass spectrometry (MS) was performed. By LMD/MS, we identified Igκ light chain with more than 95% probability but not IgD heavy chain and Igλ light chain (Fig. 2). Taken together, these observations support the diagnosis of κ-LCDD of the kidney. Although the patient did not show any evidence of portal hypertension or liver cirrhosis, but had unexplained ascites. There was a possibility that ascites could be caused by liver involvement of LCDD. Therefore, we performed a liver biopsy was performed using the CT-guided transjugular catheter technique. Liver biopsy specimens revealed the atrophy of hepatocytes and expansion of sinusoid (Fig. 3a, b). IF revealed the positive staining for anti-κ (Fig. 3c) and negative staining for anti-λ (Fig. 3d) along with the perisinusoidal membrane and vessels consisting with the involvement of κ-LCDD in the liver. Other tissue involvement included skin and salivary gland were negative. The patient was diagnosed symptomatic IgD MM and LCDD involving kidney and liver.

**Table 1:** Laboratory data on admission and on discharge the hospital

Laboratory data	On admission	On discharge	Reference ranges
Hemoglobin(g/dL)	9.5	9.2	11.0–15.3
White blood cell (/μL)	11,700	5200	3500–9800
Platelet (× 10^3^/μL)	305	214	130–370
Urine protein (dipstick)	2+	2+	Negative
Urine Sugar (dipstick)	Negative	Negative	Negative
Serum creatinine (mg/dL)	6.31	3.19	0.6–1.2
eGFR (mL/min/1.73m^2^)	5.79	12.22	> 60
Urea Nitrogen (mg/dL)	80	22.0	8.0–22.0
Uric acid (mg/dL)	10	5.0	2.6–6.0
AST (U/L)	13	8	13–33
ALT (U/L)	21	7	8–42
LDH (U/L)	330	300	124–222
Total protein	6.0	5.1	6.7–8.3
Albumin	3.8	3.8	3.4–5.8
Serum IgG (mg/dL)	282	612	680–1620
Serum IgA (mg/dL)	46	34	84–438
Serum IgM (mg/dL)	5	15	57–288
Free light chain κ (mg/L)	763	20.3	2.42–18.92
Free light chain λ (mg/L)	5.8	13.3	4.44–26.18
Free light chain κ/λ	131.55	1.526	0.248–1.804
β2-microglobulin (mg/L)	9.21	5.48	0.8–2.0
NT-proBNP (pg/mL)	3643	1068	0–125
Troponin I (pg/mL)	323.0	7.1	0–40

Abbreviations: eGFR; estimated glomerular filtration rate, AST; aspartate aminotransferase, ALT; alanine aminotransferase, LDH; lactate dehydrogenase, NT-proBNP; N-terminal prohormone of brain natriuretic peptide

Because of a rapid decline in kidney function and worsening oliguria, intermittent hemodialysis was initiated. Treatment with cyclophosphamide, bortezomib and dexamethasone (CyBorD)) followed by weekly daratumumab (16 mg/kg) was instituted. After 2 cycles of CyBorD and 8 doses of daratumumab, serum FLCκ decreased to 20.2 mg/L and the FLCκ/λ ratio normalized. Serum IFx for IgD became negative. Her serum creatinine decreased to 3.19 mg/dL after 8 infusions of daratumumab and 2 cycles of CyBorD and she became dialysis independent. Pleural effusion and ascites present at diagnosis were disappeared after treatment. The patient is received continuous treatment with daratumumab, bortezomib and dexamethasone combination with hematologic complete response.

## Discussion and conclusion

Herein, we report herein a rare case of IgD MM complicated by LCDD involving the kidney and liver. LCDD is the most frequent subtype of the group of monoclonal Ig deposition disease (MIDD) which comprises a group of disorder characterized by deposition of light chain only (LCDD), heavy chain only (HCDD), or both light and heavy chain (L/HCDD) [2]. In contrast with other renal diseases associated with M-protein (monoclonal gammopathy of renal significance, MGRS), MIDD often occurs as a complication of symptomatic myeloma. A diagnosis of MIDD requires microscopic and IF analysis, while EM is not necessary for diagnosis but is valuable for determine the pattern of deposition. Our patient presented with acute renal failure and cast nephropathy. A marked increase of FLC was the clue to the diagnosis of IgD MM and LCDD. Bone marrow biopsy showed an increase in plasma cells and serum IFx revealed a faint monoclonal IgD band. IgD level estimated from the SPEP was 230 mg/dL and serum FLCκ was elevated to 6900 mg/dL at initial presentation and decreased to 763 mg/dL 5 days later at the time of referral. Meanwhile, prednisolone (50 mg/day) was administered which appeared effective for lowering the serum FLC level but not for recovery of renal function. Renal biopsy revealed the tubular casts, nodular glomerular sclerosis and thickening of the tubular basement membrane. IF demonstrated the linear monoclonal Igκ deposition in tubular and glomerular basement membranes, indicating the diagnosis of LCDD. The EM study demonstrated the powdery deposits along the glomerular basement membrane and further confirmed the diagnosis. LMD/MS revealed the accurate diagnosis of LCDD of the kidney. A liver biopsy showed dilatation of the sinusoid and atrophy of hepatocytes. The IF study also showed the linear deposition of monoclonal Igκ along the sinusoid and vessels consistent with the diagnosis of liver involvement in LCDD. Intense linear staining of the sinusoids with Igκ was seen by immunohistochemistry. Although liver involvement of is reported to be relatively frequent in patients with LCDD, its frequency varies considerably from 0 to 19% [5, 10, 11]. In addition, the clinical scenario of LCDD patients with liver involvement has rarely been reported and most of the cases have abnormal liver function tests and/or cholestasis and are associated with high mortality [12, 13]. Our patient did not show the abnormal liver function test except for mild ascites and pleural effusion, which resolved after treatment targeted to monoclonal plasma cells. As LCDD lesions can be detected in organs other than the kidney and liver, we sought the presence of LCDD lesions in the skin and salivary glands, but there were negative for Ig deposits. Cardiac involvement was not examined considering her decreased renal function and the use of contrast media for cardiac biopsy. However, considering the presence of generalized edema and pleural effusion, and elevated NT-proBNP level, cardiac involvement of LCDD might be possible. We could not rule out the possibility of L/HCDD because of the unavailability of an appropriate anti-IgD reagent. Because liver function tests were within normal limit in this patient, it is possible that hepatic LCDD might be underestimated in patients with renal LCDD. Analysis with LMD/MS from the paraffin-embedded tissue allowed the differentiation between H/L CDD and LCDD [9]. We identified the large spectra numbers of Ig-kappa chain C region, but not that of IgD, indicating the diagnosis of LCDD, and not H/LCDD or HCDD.

Treatment with a combination of cyclophosphamide, bortezomib, dexamethasone, and daratumumab appeared effective for LCDD as well as IgD myeloma in our patient, because both FLCκ/λ normalized and serum IgD became negative by IFx and the patient became dialysis independent. The bone marrow biopsy showed a marked decrease of plasma cells to less than 1%. The patient achieved a complete hematologic response. Her ascites and pleural effusion also decreased. Although treatment of Ig deposition disease remains elusive, Sayed et al. [5] and Cohen et al. [14] reported the favorable outcomes after bortezomib based treatment in patients with LCDD. Petrakis et al. [15]reported the histologic resolution of LCDD 4 years after autologous stem cell transplantation. It seems important to obtain a deeper hematologic response for patients with LCDD, even in those with advanced kidney disease. A deeper hematologic response is a prerequisite for organ response. We used daratumumab to obtain a maximum response considering the risk of IgD MM. Daratumumab, a CD38 monoclonal antibody, has been effectively used for the treatment for the AL-amyloidosis as a single agent and combination with other anti-MM agents [16, 17]. Recently, Milani et al. [18] reported the profound hematologic response and favorable renal outcome in 6 of 7 patients with LCDD treated with daratumumab. Our patient showed significant improvement of renal function with serum creatinine levels from 6.31 mg/dl at initial admission to 3.19 mg/dl, urea nitrogen levels from 80 mg/dl to 22 mg/dl, urine protein levels from 1800 mg/day to 900 mg/day.

No serious adverse effect was observed except for mild infusion reaction. Although long-term observation is necessary for assessing the efficacy of daratumumab, the initial response seems encouraging for both hematologic and organ response in our patient.

In conclusion, we report here a patient with IgD myeloma complicated by LCDD involving the kidney and liver. Treatment with combination of novel agents, including cyclophosphamide, bortezomib, dexamethasone, and daratumumab resulted in rapid complete hematologic response and ameliorated the organ failure. We emphasize that prompt biopsy of affected organs and initiation of clone directed therapy led to the correct diagnosis and favorable outcome in patient with LCDD who has extrarenal involvement.

## Supplementary Information

**Additional file 1.** Supplemental Fig. 1 Kidney biopsy specimen showed (a) mesangial nodular glomerulosclerosis and thickened glomerular basement membrane. Mesangiolysis with aneurysmal dilatation of capillary lumen (PAS). Immunohistochemistry showed (b) positive staining for Igκand (c) negative staining for Igλ. (d) Proximal tubular basement membrane was thickened and distal tubule was occupied with PAS positive cast which was (e) positive for Igκ and (f) negative for Igλ.

## Data Availability

Not applicable.

## Abbreviations

MM: multiple myeloma
LCDD: light chain deposition disease
Ig: immunoglobulin
IF: immunofluorescence
CT: computed tomography
NT-proBNP: N-terminal pro-brain natriuretic peptide
AST: aspartate aminotransferase
ALT: alanine aminotransferase
SPEP: Serum protein electrophoresis
IFx: immunofixation
C: complement
LMD: laser microdissection
MS: mass spectrometry
CyBorD: cyclophosphamide, bortezomib and dexamethasone
HCDD: Heavy chain deposition disease
L/HCDD: light and heavy chain deposition disease
