# Supplementary figures and images for: Light chain deposition disease involving kidney and liver in a patient with IgD myeloma
Source: BMC Nephrol. 2021 Jan 23;22:40. doi: 10.1186/s12882-021-02246-9 (PMC7824927; doi:10.1186/s12882-021-02246-9)

Supplemental Figure 1

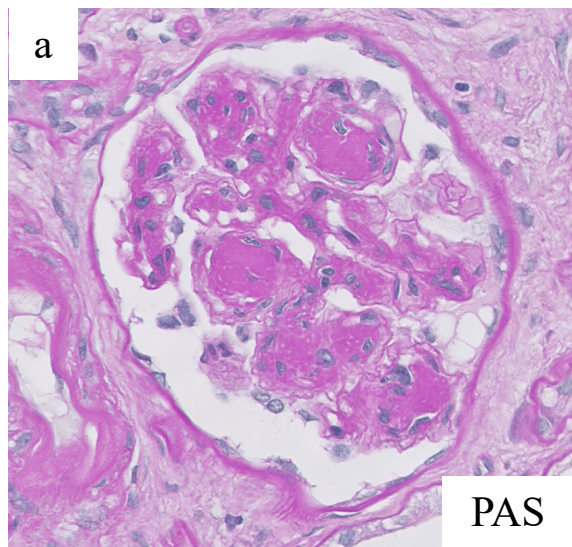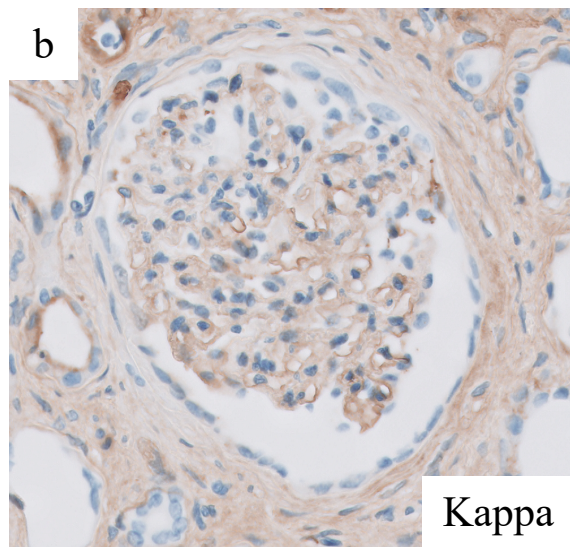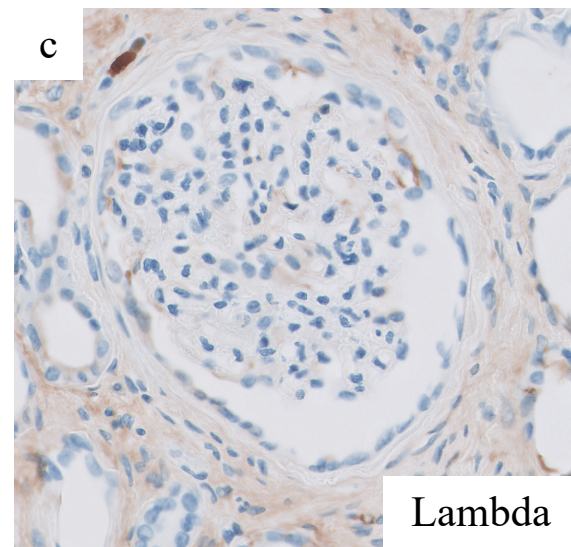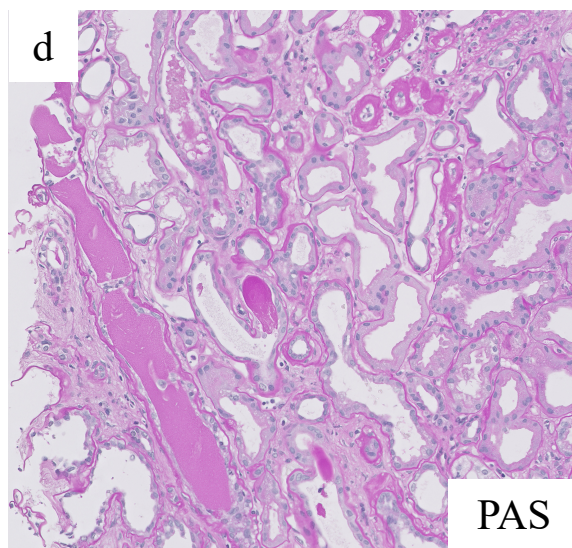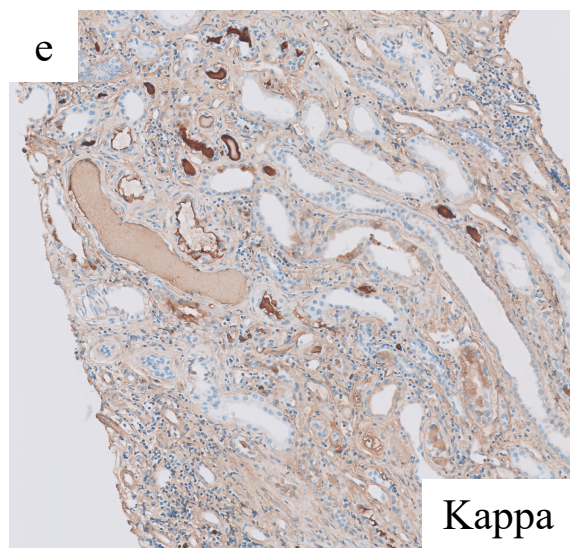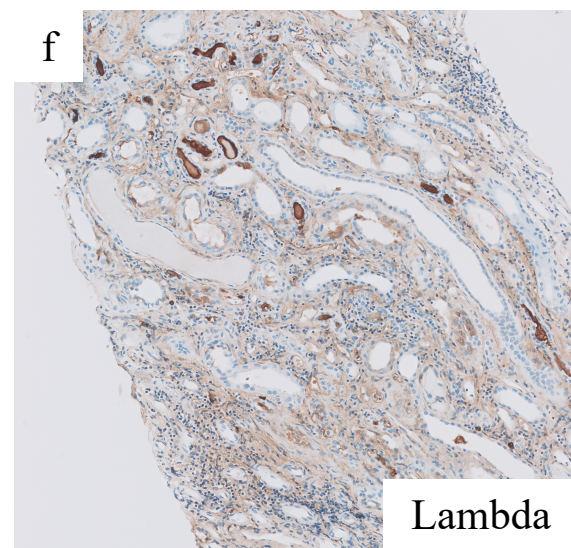

Supplement: Supplementary file 1 — Additional file 1. Supplemental Fig. 1 Kidney biopsy specimen showed (a) mesangial nodular glomerulosclerosis and thickened glomerular basement membrane. Mesangiolysis with aneurysmal dilatation of capillary lumen (PAS). Immunohistochemistry showed (b) positive staining for Igκand (c) negative staining for Igλ. (d) Proximal tubular basement membrane was thickened and distal tubule was occupied with PAS positive cast which was (e) positive for Igκ and (f) negative for Igλ. [file 12882_2021_2246_MOESM1_ESM.pdf]
